# Current Opinion on Molecular Characterization for GBM Classification in Guiding Clinical Diagnosis, Prognosis, and Therapy

**DOI:** 10.3389/fmolb.2020.562798

**Published:** 2020-09-08

**Authors:** Pei Zhang, Qin Xia, Liqun Liu, Shouwei Li, Lei Dong

**Affiliations:** ^1^School of Life Sciences, Beijing Institute of Technology, Beijing, China; ^2^Department of Neurosurgery, Sanbo Brain Hospital, Capital Medical University, Beijing, China

**Keywords:** glioblastoma, molecular heterogeneity, transcription-based subtype, genetic alteration-based subtype, DNA methylation-based subtype, subtype-specific therapy

## Abstract

Glioblastoma (GBM) is highly invasive and the deadliest brain tumor in adults. It is characterized by inter-tumor and intra-tumor heterogeneity, short patient survival, and lack of effective treatment. Prognosis and therapy selection is driven by molecular data from gene transcription, genetic alterations and DNA methylation. The four GBM molecular subtypes are proneural, neural, classical, and mesenchymal. More effective personalized therapy heavily depends on higher resolution molecular subtype signatures, combined with gene therapy, immunotherapy and organoid technology. In this review, we summarize the principal GBM molecular classifications that guide diagnosis, prognosis, and therapeutic recommendations.

## Introduction

The World Health Organization (WHO) defines adult diffuse gliomas into grade II and grade III astrocytic tumors, grade II and III oligodendrogliomas, and grade IV glioblastomas (Louis et al., 2016). Glioblastoma (GBM) is grade IV, the most invasive and deadly glioma (Brennan et al., 2009; Szopa et al., 2017; Lee et al., 2018; Ghosh et al., 2018; Shergalis et al., 2018; Paolillo et al., 2018). It invades adjacent areas of the brain but rarely spreads outside the brain (Phillips et al., 2006). Clinical data show GBM has a poor prognosis, with less than 5% of patients surviving 5 years after diagnosis (Verhaak et al., 2010). Based on clinicopathologic features, GBM is defined as primary or secondary GBM (Ohgaki and Kleihues, 2013). Primary GBM starts as grade IV, with no evidence of lower grades, and is more aggressive and more likely to affect elderly patients. Secondary GBM develops from astrocytoma (Grade II or III glioma), grows slowly initially then gradually becomes aggressive (Ohgaki and Kleihues, 2007). The mechanism of GBM tumorigenesis is still unclear, many patients relapse due to ineffective treatment options. Notably, recurrent GBM is frequently accompanied by molecular alterations compared with the initial diagnosis (Li et al., 2015; van den Bent et al., 2015; Cioca et al., 2016; Neilsen et al., 2019; Schafer et al., 2019).

Histomorphology ambiguity and tumor heterogeneity pose challenges to GBM diagnosis, prognosis and treatment. Histologic diagnosis often varies among clinicians and limits diagnostic reproducibility. GBM histologically and genetically show significant inter-tumoral and intra-tumoral heterogeneity, differing mutations, and indistinct phenotypic and epigenetic states reflect genomic instability that leads to varying therapy choices and clinical outcomes (Homma et al., 2006; Marusyk and Polyak, 2010; Szerlip et al., 2012; Brennan et al., 2013). Molecular classification of GBM is a newer tool and a complement to the traditional pathology-based description (Verhaak et al., 2010; Brennan et al., 2013; Ceccarelli et al., 2016).

Molecular-based diagnosis, patient stratification, and personalized treatment are increasingly important. The ISN Haarlem recommends “hierarchical diagnosis with histological classification, WHO classification, and molecular information for comprehensive diagnosis” (Louis et al., 2014). In 2016, the WHO updated guidelines combining morphology and genetic variation, leading to a significant reorganization of the classification of several brain tumor entities, especially in gliomas (Louis et al., 2016). Two significant entities of 2016 WHO classification based on IDH (Isocitrate dehydrogenase) gene mutant status are IDH wild-type and IDH mutated GBM; patients whose full IDH evaluation cannot be assessed are classified as GBM NOS (not otherwise specified) (Louis et al., 2016).

Multi-omics studies from the landscape of GBM in the Cancer Genome Atlas Research Network (TCGA), the Chinese Glioma Genome Atlas (CGGA), and other databases, together reveal the complicated genetic profile of GBM (Cancer Genome Atlas Research Network, 2008; Brennan et al., 2013; Zhao Z. et al., 2020). These aberrant molecules, including 1p and 19q co-deletions (oligodendroglioma-specific), IDH gene mutations, PTEN (Phosphatase and tensin homolog) gene mutations, TP53 mutations, TERT (Telomerase reverse transcriptase) gene promoter mutations, ATRX (Alpha thalassemia/mental retardation syndrome X-linked) gene mutations, and EGFR (Epithelial growth factor receptor) gene amplification, are forcing clinicians to reconsider traditional GBM treatment (Mclendon et al., 2008; Brennan et al., 2013). GBM classification based on aberrant molecules shortens the time from diagnosis to treatment, and significantly improves accuracy and targeting.

In this paper, we summarize the process of GBM classification based on transcription levels, genetic alterations, and DNA methylation. We also describe the molecular characteristics of each category, and the relationship between different classification methods. Finally, we provide the current guiding strategy for diagnosis and treatment.

## GBM Heterogeneity Identified by Transcription, Genetic Alteration, and DNA Methylation

Deciphering GBM heterogeneity and complexity is the key to understanding it’s progression and creating effective therapies. Some important and aberrant molecular events drive GBM malignant transformation, highlighting the importance of molecular classification. First, GBM has a wide variety of chromosomal changes, including amplification in chromosome 4 (Chr.4, PDGFRA), Chr.7 (EGFR; MET, hepatocyte growth factor receptor; CDK6, Cyclin-dependent kinase 6), Chr.12 (CDK4, Cyclin-dependent kinase 6; MDM2, Mouse double minute 2 homolog), and deletion in Chr.10 (PTEN). Notably, some GBM patients have simultaneous gain of Chr.19 and 20 (Brennan et al., 2013).

Second, the TCGA GBM project describes somatic genome changes based on multidimensional and comprehensive features that show significant mutations in GBM, including TP53 (34.4%), EGFR (32.6%), PTEN (32%), NF1 (Neurofibromin 1, 13.7%), PIK3CA (Phosphatidylinositol 4,5-bisphosphate 3-kinase catalytic subunit alpha isoform, 12%), PIK3R1 (Phosphatidylinositol 3-kinase regulatory subunit alpha, 11.7%), RB1 (Retinoblastoma-associated protein 1, 9.3%), SPTA1 (Spectrin alpha chain, erythrocytic 1, 9%), ATRX (6%), IDH1 (5.2%), KEL (Kell blood group glycoprotein, 5%), PDGFRA (Platelet-derived growth factor receptor A, 4.5%), and GABRA6 (Gamma-aminobutyric acid receptor subunit alpha-6, 4%) (Cancer Genome Atlas Research Network, 2008; Parsons et al., 2008; Verhaak et al., 2010; Brennan et al., 2013).

Lastly, DNA methylation is a key factor when measuring heterogeneity and stratification of GBM patients. Epigenetic modifications of GBM is related to biological characteristics and are considered therapeutic targets (Hegi et al., 2005; Etcheverry et al., 2010; Romani et al., 2018; Carella et al., 2020). DNA methylation states in GBM are correlated with survival, which has been extensively explored in recent years (Lofton-Day and Lesche, 2003; Hegi et al., 2005; Etcheverry et al., 2010; Christensen et al., 2011). GBM genome-wide methylation data show biologically distinct subtypes (Brennan et al., 2013). For example, DNA methylation of the MGMT (O6-Methyl guanine DNA methyltransferase) gene promoter occurs in 48.5% of GBM patients (174/359); MGMT is a known marker for treatment strategy (Parsons et al., 2008). Additionally, GBM patient data show other methylated genes, including GATA6 (GATA binding protein 6) (68.4%), CD81 (CD81 antigen) (46.1%), DR4 (Death receptor 4) (41.3%) and CASP8 (Caspase-8) (56.8%) (Skiriute et al., 2012). Interestingly, H. Noushmehr et al. found CpG island hypermethylation in a distinct subgroup of gliomas (G-CIMP), however only a small number of GBM patients with a positive prognosis belong to G-CIMP phenotype (Noushmehr et al., 2010).

## Molecular-Based GBM Classification in Diagnosis and Prognosis Prediction

With the recent development of technology and classification algorithms, GBM is divided into different subtypes based on transcription profiles, genetic alterations, and DNA methylation. This allows targeted therapy based on molecular characteristics of subclasses. For example, clinicians can target the mesenchymal subtype from transcription subtypes in GBM via inhibition of diacylglycerol kinase alpha. In doing so, patients with MGMT methylation had a more robust response to temozolomide (Hegi et al., 2005; Taylor and Schiff, 2015; Olmez et al., 2017). The TCGA GBM project used a multi-platform analysis and comprehensively determined the genomic landscape to better understand the pathogenic and drug-resistant mechanism of GBM (Brennan et al., 2013). Here, we describe the classical classification, and analyze the differences among various GBM subtypes.

### Transcription-Based Subtypes

**FIGURE 1:**
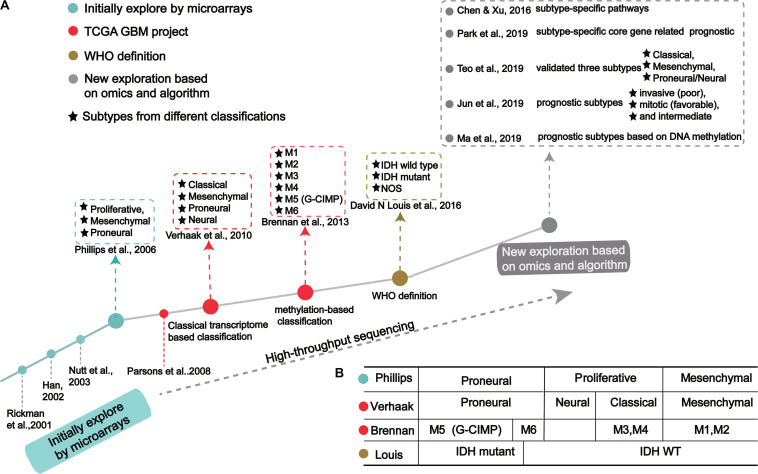
The process of molecular-based GBM classification: **(A)** GBM classification timeline and classical subtypes. **(B)** The relative overlap between subtypes from different classification methods.

GBM classification based on gene expression profiles initially used microarray technology, then large-scale high-throughput next-generation sequencing technology. The molecular map of GBM is shown in Figure 1A. The classification method proposed by Verhaak et al. has been widely used, includes four subtypes: Proneural, Neural, Classical and Mesenchymal (Verhaak et al., 2010).

#### Initial Exploration on the Transcription-Based Classification

In the 1990s, scientists acquired data from techniques like PCR, allele analysis, and first-generation sequencing to analyze gliomas. They found a variety of molecular markers of different types and grades, but the landscape was not clear (Sehgal, 1998). Indeed, tumor development is highly complex, involving multiple genetic and epigenetic changes. Through microarray investigations, genes associated with GBM were identified and used as biomarkers in early diagnosis, leading many researchers to begin exploration of molecular diagnosis, classification, and treatment (Figure 1A; Schena et al., 1995; Velculescu et al., 1995; Derisi et al., 1996; Dudoit et al., 2002; Irizarry et al., 2003; Hu et al., 2006). Rickman et al. found 360 distinct genes in GBM from pilocytic astrocytomas, including MDM2, IGFBP2 (Insulin-like growth factor-binding protein 2), CD44 (CD44 antigen), and CDK4 (Cyclin-dependent Kinase 4) (Rickman et al., 2001). Sallinen et al. found more than 200 gene expression alterations in GBM and demonstrated a strategy for high-throughput molecular genetic profiling of brain tumors (Sallinen et al., 2000). In addition, Nutt et al. found 14 GBMs and 7 anaplastic oligodendroglioma, diagnosed by pathology, were predicted using gene markers that accurately classified 18 samples (Nutt et al., 2003). The classification prediction model objectively and reliably classifies high-grade non-classical glial tumors (Nutt et al., 2003). Compared with pathological classification, this model reliably predicts the prognosis of atypical lesions more accurately.

In a groundbreaking study, Phillips et al. classified three GBM subtypes: Proneural, Proliferative and Mesenchymal (Figure 1 and Table 1; Phillips et al., 2006). Proneural subtypes are more common in young patients, less pathological compared with proliferative or interstitial GBM and have a better prognosis (Phillips et al., 2006). NCAM (Neural cell adhesion molecule), GABBR1 (Gamma-aminobutyric acid type B receptor subunit 1), and SNAP91 (Clathrin coat assembly protein AP180) are associated with neurons and are more similar to normal brain tissue and expression in proneural subtype (Phillips et al., 2006). The Proliferative subtype is similar to stem cells with significantly up-regulated markers of proliferation, including TOP2A (DNA topoisomerase II alpha) and PCNA (Proliferating cell nuclear antigen) (Phillips et al., 2006). In contrast, the Mesenchymal subtype displays overexpression of angiogenesis markers, including the endothelial marker PECAM1 (Platelet endothelial cell adhesion molecule) gene, VEGF (Vascular endothelial growth factor) gene, VEGFR1 (Vascular endothelial growth factor receptor 1) gene and VEGFR2 (Vascular endothelial growth factor receptor 2) gene, which shows mesenchymal and angiogenic characteristics (Phillips et al., 2006). Proliferative and Mesenchymal subtypes are characterized by activation of PI3K/AKT (Phosphoinositide 3-kinase/Protein kinase B) signaling, loss on Chr.10 (location of PTEN), gain on Chr.7 (location of EGFR), and poor prognosis with invasive growth and angiogenic pathways (Phillips et al., 2006). These three subtypes are reminiscent of the various stages of developmental neurogenesis, which provides the basis and perspective for the molecular classification of GBM.

**TABLE 1:** The classification by Phillips, Verhaak and Wang.

**Phillips et al. (2006)**		**Proneural**	**Proliferative**		**Mesenchymal**
	Signature	NCAM, GABBR1, SNAP91	PCNA, TOP2A, EGFR		VEGF,VEGFR1, VEGFR2, PECAM1
	Chromosome Gain/loss	None	Gain on Chr.7, loss on Chr.10		Gain on Chr.7, loss on Chr.10
	Biological process	Neurogenesis	Proliferation		Angiogenesis

**Verhaak et al. (2010)**		**Proneural**	**Neural**	**Classical**	**Mesenchymal**

	Signature	PDGFRA, OLIG2, DDL3,SOX2, NKX2-2	MBP/MAL, NEFL, SLC12A5, SYT1, GABRA1	EGFR, AKT2, SMO, GAS1, GLI2, NOTCH3, JAG1, LFNG	YKL40, MET, CD44, MERTYK, TRADD, RELB, TNFRSF1A
	Mutated genes	TP53, PI3K, IDH1, PDGFRA		PTEN, CHKN2, PDGFRA	NF-κB, NF1

**Wang et al. (2017)**		**Proneural**	**Neural**	**Classical**	**Mesenchymal**

	Cell source	Tumor cells	Tumor cells	Tumor cells	Non-tumor cell

#### Deep Analysis of Transcription-Based Classification

The above studies demonstrated tumors often cluster in groups that display heterogeneity, highlighting the weaknesses of conventional diagnosis. With the advent of large-scale, high-throughput, next-generation sequencing methods, and with algorithms in machine learning, complex tumor data is becoming more precise.

Verhaak et al. (2010) offered more in-depth research and treatment possibilities for GBM (Figure 1A) based on the four subtypes Proneural, Neural, Classical and Mesenchymal (Table 1). The Proneural subtype is found primarily in younger patients, characterized by high PDGFRA gene expression and frequent IDH1 mutation. Compared with the other three subtypes, the Proneural subtypes may have better survival rates. However, Proneural subtypes showed no significant difference from other subtypes in response to chemotherapy and radiotherapy (Colman et al., 2010). The Neural subtype has similar gene expression patterns compared with normal brain tissue and tends to be more responsive to radiation and chemotherapy. GBMs with neural markers like SYT1 (Synaptotagmin 1), SLC12A5 (Solute carrier family 12 members 5), GABRA1 (Gamma-aminobutyric acid type A receptor alpha1) and NEFL (Neurofilament light polypeptide), are classified as the Neural subtype. The Classical subtype shows aberrant changes, including Chr.7 amplification, Chr.10 loss, inactivation of the RB (Retinoblastoma-associated protein) pathway, and focal 9p21.3 homozygous deletion. In addition, Sonic hedgehog pathways (SMO, Smoothened homolog; GAS1, Growth arrest-specific protein 1; GLI2, Growth arrest-specific protein 2), Notch signaling pathways (NOTCH3, Neurogenic locus notch homolog protein 3; JAG1, Jagged1; LFNG, Lunatic fringe) and the neural precursor and stem cell marker NES are highly expressed in the Classical subtype. Importantly, patients with Classical subtype show a significant reduction in mortality with aggressive radiotherapy and chemotherapy. The Mesenchymal subtype is characterized by extensive necrosis and inflammation, upregulation of interstitial and angiogenesis genes, deletion of tumor suppressor genes P53, PTEN, and NF1, and high expression of genes in the tumor necrosis factor superfamily and the NF-κB pathway. Although responsive to aggressive radiotherapy and chemotherapy, the prognosis of Mesenchymal subtypes is the worst among all subtypes (Colman et al., 2010). Recently, Sharma et al. found that VEGF-A (Vascular endothelial growth factor A), VEGF-B (Vascular endothelial growth factor B), ANG1 (Angiopoietin 1) and ANG24 (Angiopoietin 24) genes are highly expressed in the Mesenchymal subtype (Sharma et al., 2017).

In 2017, Wang et al. (2017) proved that GBM tumor cells include Classical, Proneural, and Mesenschymal, and Neural subtype is non-tumor cells in the tumor microenvironment. They found the median survival of Mesenchymal, Classical, or Proneural are 11.5, 14.7, and 17.0 months, respectively. Wang’s classification is based on tumor cells rather than microenvironmental/non-malignant tumor cells in tumor entities.

Using cancer genome data from the TCGA GBM project and classification from Verhaak et al. (2010) and Park A. K. et al., 2019 identified subtype-specific prognostic core genes and further examined prognostic chromosome changes and mutations (Figure 1A). Specific prognostic core genes in Classical subtype exist in DNA repair, cell cycle, Janus kinase, and transcription activation factor (JAK-STAT) pathway. And, specific prognosis genes in Mesenchymal subtype are related to mesenchymal cell movement, PI3K/AKT pathway, Mitogen-activated protein kinase (MAPK) pathways, extracellular signal-regulated kinase (ERK) pathways, and Wnt pathways (Park A. K. et al., 2019). Notably, patients with Mesenchymal subtypes with PIK3R1 or PCLO (Protein piccolo) mutations show a poorer prognosis (Park A. K. et al., 2019). These results demonstrate specific molecular targets and biomarkers for each subtype of GBM.

Recent studies offer new insights into GBM classification based on transcription. Teo et al. validated three robust GBM-subtypes: Proneural/Neural, Classical, and Mesenchymal across six different datasets (Figure 1A; Verhaak et al., 2010; Teo et al., 2019). This was validated in subtype-specific patient-derived orthotopic xenograft (PDOX) mice; the Classical subtype showed no survival difference between radiotherapy and temozolomide monotherapy. A Proneural/Neural specific-PDOX model showed temozolomide significantly improved survival compared to radiotherapy. This points to better predictive clinical outcomes based on more precise patient selection in clinical trials.

Park J. et al. (2019) identified three subtypes related to prognosis prediction: Mitotic (favorable), Intermediate, and Invasive (poor) by analyzing and verifying four large-scale gene expression profiles (Figure 1A). These new GBM subtypes have different multi-omics features and biological phenotypes. Among GBM prognostic subtypes, the invasiveness in the Invasive subtype is significantly higher than the Mitotic subtype. Interestingly, the methylated MGMT gene promoter is correlated with the Mitotic subtype, indicating Mitotic subtype patients are more likely to respond to temozolomide (Park J. et al., 2019). This study suggests that treatment strategies should be based on prognostic subtypes. For example, patients in the Mitotic subtype can be treated with temozolomide, while patients in Invasive subtypes require therapeutic intervention for the aggressiveness of the GBM. Although the prognostic subtype is based only on transcription and survival time, genomic features such as pathogenic somatic variations of IDH1 and ATRX and DNA methylation are only present in Mitotic subtypes. Since these three subtypes suggest a prognosis for GBM, inhibition of target genes in different subtypes may improve patient survival. Further, these genes may have clinical value as prognostic biomarkers and new drug targets, while also leading to new pathological and etiological factors for the oncogenesis and development for GBM.

### Genetic Alteration-Based Subtypes

In recent years, large-scale genomic studies have revealed many mutations in tumor suppressor genes and oncogenes, and significantly improved our understanding of GBM. Specifically, mutated IDH, PTEN and EGFR are related to patient survival and can be used as indicators of patient classification.

#### IDH-Wild Type and IDH-Mutation Type

The identification of the IDH mutation is an important contribution to the molecular pathology of GBM. In 2008, Parsons et al. (2008) found the IDH1 gene had a point mutation in a small number of glioblastoma samples. Subsequently, Yan et al. (2009) found that GBM patients with IDH1/IDH2 mutations had a higher survival rate than those without these mutations (Table 2). Many studies have shown that patients with IDH mutations are significantly different from those without IDH mutations in molecular and clinical characteristics, including prognosis (Ichimura et al., 2009; Nobusawa et al., 2009; Watanabe et al., 2009; Yan et al., 2009; Lu et al., 2012; Songtao et al., 2012; Stancheva et al., 2014; Mondesir et al., 2016). There are three IDH enzymes: IDH1, IDH2, and IDH3 (Yan et al., 2009). IDH1 is mainly cytoplasmic, while IDH2 and IDH3 are mostly present in the mitochondrial matrix. IDH is the central enzyme in the citric acid cycle and plays a vital role in oxidative stress resistance (Marko and Weil, 2013). The most common IDH1 mutation observed in gliomas is the point mutation at position 132 (R132H), which is regarded as a typical IDH1 mutation (Parsons et al., 2008).

**TABLE 2:** The characteristics of IDH WT subtype and IDH mutant subtype.

	**IDH WT**	**IDH mutant**	**References**
Corresponds to	Primary GBM	Secondary GBM	Louis et al., 2016
Proportion	90%	∼10%	Louis et al., 2016
Age	Usually > 60	Younger adults	Louis et al., 2016
CpG methylator	Less frequent	More frequent	Brennan et al., 2013
TERT promoter mutation	∼95%	51%	Yan et al., 2009
homologous deletion of CDKN2A/CDKN2B	∼45%	Less	Yan et al., 2009
EGFR alterations	∼41%	0%	Yan et al., 2009
PTEN mutation/deletion	∼25%	0%	Yan et al., 2009
TP53 mutations	∼20%	81%	Yan et al., 2009

In 2016, the WHO divided it into two: IDH mutation and IDH wild type (Figure 1A; Louis et al., 2016). IDH wild type GBM with poor survival is dominated by stellate cell differentiation, characterized by nuclear atypia, cell polymorphism, typical diffuse growth patterns, mitotic activity and microvascular proliferation and/or necrosis. There are three variants of IDH wild-type, including giant cell GBM, gliosarcoma and epithelial-like GBM (Ep-GBM) (Louis et al., 2016). Genetically, giant cell GBM lacks EGFR amplification and homozygous CDKN2A deletion and contains PTEN mutation and TP53 mutation (Meyer-Puttlitz et al., 1997). Patients with the giant cell GBM have outcomes similar to classical GBM. In gliosarcoma, TP53 mutations are rare, and EGFR amplification is also uncommon, and contains CDKN2A deletion (Lowder et al., 2019). The clinical outcome of gliosarcoma differs from classical GBM, but there are still conflicting and uncertain results from various studies. Ep-GBM, as a new variant of GBM, is more prevalent in children and young people, manifesting as superficial brain or mesencephalic masses, and often carries BRAF (Serine/threonine-protein kinase B-Raf) V600E mutations (Chapman et al., 2011; Kleinschmidtdemasters et al., 2013; Broniscer et al., 2014). Ep-GBM is based on the absence of INI1 expression, distinguishing it from similar epithelioid counterparts (Kleinschmidt-DeMasters et al., 2010). Additionally, Ep-GBM often lacks EGFR amplification and PTEN loss, but ODZ3 usually has hemizygous deletions (Alexandrescu et al., 2016).

Multiple studies have confirmed that IDH mutations have prognosis and predictive value (Yan et al., 2009; Beiko et al., 2014; Stancheva et al., 2014). Compared to GBM patients with wild-type IDH, IDH-mutant GBM patients had higher overall survival and were more responsive to temozolomide (Songtao et al., 2012). The inhibitor of IDH mutation, which has been applied in preclinical models, shows activity to retard glioma cell growth (Rohle et al., 2013).

#### Other Genetic Mutations

In IDH1 wild type GBM, the median survival rate of patients with CDK4/MDM2 co-amplification is 6.6 months after diagnosis, while the median survival rate of patients without an CDK4/MDM2 co-amplification is 12.7 months (Abedalthagafi et al., 2018). The TERT promoter mutation was recently identified as a sign of poor prognosis. It is enriched in elderly patients, with approximately 40% having grade II/III glioma, suggesting TERT’s correlation with shorter overall survival as a key pathological player and therapeutic target (Chamberlain and Sanson, 2015; Mosrati et al., 2015; Spiegl-Kreinecker et al., 2015; Yang et al., 2016; Yuan et al., 2016).

EGFR amplification is usually accompanied by EGFR mutation, the most frequent being EGFRvIII (Gan et al., 2009). Under normal physiological conditions, EGFR plays a central role in cell proliferation, differentiation and development. EGFR is located on the short arm of Chr.7 (7p12) and encodes a cell surface receptor tyrosine kinase (Hatanpaa et al., 2010). EGFRvIII is characterized by the absence of 267 amino acids in the extracellular domain, resulting in the inability of the receptor to bind to the ligand but with substitutive activity (Hatanpaa et al., 2010). EGFRvIII enhances the tumorigenic potential of GBM by activating and maintaining mitotic and anti-apoptotic signaling pathways, along with their impaired internalization and degradation (Gan et al., 2009). Some studies have found that EGFRvIII overexpression and EGFR amplification are associated with poor prognosis in young patients, and other data show EGFR overexpression is associated with poor prognosis in elderly patients (Shinojima et al., 2003; Srividya et al., 2010). But recently, Felsberg et al. found EGFRvIII and EGFR SNVs are not prognostic; Chen et al. showed that there is insufficient evidence for the presence of either EGFR amplification or EGFRvIII mutation has prognostic value in patients with GBM using meta-analysis (Chen et al., 2015; Felsberg et al., 2017). These results may be biased by the inherent variability in subtypes, therefore, the exploration of the relationship between EGFR and prognosis needs to be carried out in different subtypes. Notably, compared with patients with both TERT and EGFR gene mutations, the overall survival of TERT/EGFR wild-type patients (EGFR not amplified) is almost twice that of the former (Chamberlain and Sanson, 2015; Yang et al., 2016).

The PTEN protein catalyzes the dephosphorylation of 3’ phosphorylation of the inositol ring in PIP3 (phosphatidylinositol-3,4,5-trisphosphate) to produce PIP2 (phosphatidylinositol-4,5-bisphosphate). The dephosphorylation is critical because it inhibits the AKT signaling pathway. The PI3K/AKT pathway is normally dormant in differentiated and quiescent cells, but when activated, the cell cycle modulation leads to cancer. The deficiency of PTEN mainly plays the role of lipid phosphatase through the PI3K/AKT pathway (Endersby and Baker, 2008). Therefore, the loss of PTEN is associated with a more aggressive phenotype.

In addition to the genes above, other genetic mutations also drive GBM development. However, the mutation or deletion of a single gene may not serve to classify GBM independently. The combination of aberrant events related to survival may be a more effective classifier. Data suggest combination of two or three genes provide a robust classifier to diagnostic analysis for clinical applications (Kim et al., 2002). Classification driven by genetic mutation (single or consistent) is the basis for exploring GBM classification, and critical genetic targets can be used as the key for diagnosis, prognosis and treatment.

### DNA Methylation-Based Subtypes

Epigenetic changes are common markers of human cancers, including GBM (Kim and Kim, 2008; Romani et al., 2018). DNA methylation is a core element of epigenetic alteration, an essential signaling tool for regulating genomic functions, and a key feature mediating tumorigenesis (Koch et al., 2018; Muhammad et al., 2018; Yamashita et al., 2018). DNA methylation can provide biomarkers for the early diagnosis and prognosis of cancer and provide a new method for further clinical applications (Lofton-Day and Lesche, 2003; Gustafsson et al., 2018; Kim et al., 2018; Li et al., 2018, 2019; Pérez et al., 2018. We know the methylation status of single genes corresponds to expression levels in GBM (Bell et al., 2018; Johannessen et al., 2018). MGMT promoter methylation is a prognostic factor for glioblastoma patients and has a significant correlation with worse survival rates (16.9 months vs. 12.7 months) (Brennan et al., 2013). Due to the diversity of GBM, a broader genome and expression profile is needed to gain insight into the potential response of treatment methods.

Brennan et al. used large-scale methylated sequencing data to classify GBM, divided into six categories based on the expression level of DNA methylation, including Cluster M1 to Cluster M6, in which Cluster M5 was G-CIMP subtype (Figure 1A; Brennan et al., 2013). Cluster M6 is relatively hypomethylated and has the majority of IDH1 wild type patients than the G-CIMP subtype. Cases of missense mutations or deletions in MLL (Histone-lysine N-methyltransferase 2A) genes or HDAC (Histone deacetylase) family genes were concentrated in Cluster M2 (Brennan et al., 2013). These results indicate that the classification based on DNA methylation makes GBM classification clearer.

Recently, Ma et al. (2019) identified specific prognostic subtypes based on DNA methylation status and identified 3 GBM methylation clusters (Cluster 1, Cluster 2, and Cluster 3), which have significantly different survival curves (Figure 1A). Among all clusters, Cluster 2 has the best prognosis. The methylation levels in each cluster are related to specific molecular characteristics. Compared with Cluster1 and Cluster2, Cluster 3 showed more TP53 mutations and deletion of wildtype IDH1 and 1p/19q. The genes corresponding to the promoter region of the CpG site annotation are related to the survival and biological processes in GBM. By focusing on the level of DNA methylations in patients with GBM, researchers eventually developed a new prediction panel for 10 CpGs. They are superior to other molecular indicators because these 10 CpG signals reflect the relationship between GBM intrinsic tumor subtypes (Kloosterhof et al., 2013; Paul et al., 2017; Yin et al., 2018). The study also found the enriched CpG sites in genes involved in neuronal differentiation and brain development, including KIFC3 (Kinesin-like protein), OC90 (Otoconin-90), CRB2 (DNA repair protein crb2), IGSF22 (Immunoglobulin superfamily member 22) and NR0B2 (Nuclear receptor subfamily 0 group B member 2) (Wu et al., 2010; Yu et al., 2013).

DNA methylation provides a framework for understanding GBM and guiding a therapeutic strategy. It has offered more molecular biomarkers for each subtype and suggested more targets for treatment. Methylation is a powerful complement to classification based on genetic alterations and transcription, making GBM classification more comprehensive.

### The Relationship Among Transcription, Genetic Alterations and DNA Methylation Classifications

Early attempts to identify specific tumor subtypes generally focused only on gene expression patterns. But biological processes are not so simply regulated. Omics data have helped identify clusters of tumors with similar characteristics, including genotypic and epigenetic regulation. Many studies have found that molecular subtypes classified at different levels are related and overlapped, as exampled from the four transcription-based subtypes from Verhaak et al. (2010), six DNA methylation-based subtypes from Brennan et al. (2013) and IDH mutation-based subtypes (Figure 1B). Combined analysis with four transcriptome-based subtypes of TCGA, Cluster M1 and M2 are enriched in the Mesenchymal subtypes, Cluster M3 and M4 in the Classical subtype, Cluster G-CIMP in the Proneural subtype, and Cluster M6 is relatively hypomethylated, which belongs to the Proneural subtype (Verhaak et al., 2010). Notably, Cluster G-CIMP increases the likelihood of DNA methylation of MGMT (79% of patients with DNA methylation of MGMT in Cluster G-CIMP and 46% in non-G-CIMP). Interestingly, MGMT DNA methylation is a predicted biomarker of classical subtypes, but not other subtypes. In addition, C-CIMP is a unique and almost invariable feature of IDH1/2 mutant GBMs, and studies have shown that patients with this GBM subtype have a better prognosis (Noushmehr et al., 2010; Baysan et al., 2012). According to the characteristic of DNA methylation pattern causally related to IDH1/2 mutation status and better prognosis, the Proneural subtype is further subdivided into G-CIMP positive and negative groups (Noushmehr et al., 2010).

## Molecular Subtype Migration in Recurrent GBM

Recently, some studies have shown that subtype migration and molecular changes occur in recurrent GBM, highlighting the need for further research (Wang et al., 2016). The recurrence of GBM is inevitable, although current standards of care for GBM patients include chemotherapy after surgical resection (Stupp et al., 2005). However, when GBM occurs, the tumor always recurs, and treatment options are limited. There is no standard care for patients with relapsed GBM because pathological and molecular features are lacking (Weller et al., 2012; Lukas and Mrugala, 2016). The progression-free survival of recurrent GBM is 2–4 months, and the survival of conventional chemotherapy after progression is 6–8 months (Gorlia et al., 2012).

Transcription-based molecular subtypes are also associated with tumor recurrence. For example, Wang et al. found that two-thirds of patients with primary GBM switched transcriptional subtype after recurrence. Importantly, the Mesenchymal subtype was the most stable primary GBM subtype (Wang et al., 2016). Therefore, further analysis of the molecular changes of recurrent GBM poses significant value in guiding treatment. van den Bent et al. showed that half of recurrent GBM patients lost EGFRvIII compared with the molecular expression of GBM at initial diagnosis (Table 3; van den Bent et al., 2015). Cioca et al. found recurrent GBM had lower EGFR expression than primary GBM in 10 cases, and only one case had increased expression on recurrence (Table 3; Cioca et al., 2016). The discrepancies of EGFR expression between primary GBM and recurrence suggest heterogeneity of GBMs is actively fluid. Neilsen et al. analyzed 10 pairs of matched primary and recurrent GBM through genomic changes, and the results indicate all matched tumor pairs showed differences. This study showed that EGFR mutation increased significantly in 3 cases, and the other three genes were generally changed in primary GBM and recurrent GBM, namely CDKN2A and CDKN2B deletion, and TERT mutation. Mutations that cause activation of the PI3K pathway are also common (Table 3; Neilsen et al., 2019). Kim et al. (2015) found that recurrent GBM had a hypermutant phenotype that initially occurred in the IDH1 mutant, suggesting IDH1 is associated with a hypermethylated phenotype, resulting in MGMT inhibition, making tumors more susceptible to mutagenesis by temozolomide.

**TABLE 3:** The molecular changes in recurrent GBM.

**Event**	**Primary vs. Recurrent**		**References**
EGFRvIII	About half of recurrent GBM patients lose EGFRvIII (7/15)		van den Bent et al., 2015

EGFR	Lower EGFR expression at recurrent GBM		Cioca et al., 2016

	**Initial**	**Recurrent**	

CDKN2A deletions	86%	53%	Neilsen et al., 2019
CDKN2B deletions	86%	54%	
EGFR mutation	52%	10%	
EGFR amplification	81%	45%	
TERT mutation	95%	51%	

Studies focused on recurrent GBM have shown molecular composition and molecular subtypes of tumors evolve in response to radiotherapy and targeted therapy, therefore molecular signatures guiding treatment protocols may improve patient survival (Campos et al., 2016). However, it is still challenging to develop new molecular therapies for recurrent GBM patients and personalized treatment.

## Molecular Subtypes and Signatures Guiding Clinical Treatment

### Subtype-Specific Molecular Guidance for the Selection of Targeted Drugs

Treatment corresponding to tumor subtypes is an effective strategy to avoid the obstacles caused by molecular heterogeneity (Collisson et al., 2011; Linnekamp et al., 2015; Zhao S. et al., 2020). Chen et al. analyzed the relationship between four subtypes distinguished by Verhaak (Figure 1A; Verhaak et al., 2010; Chen and Xu, 2016). The gene signatures in the Mesenchymal subtype is highly enriched in pathways associated with immune response, such as Hepatic Fibrosis/Hepatic Stellate Cell Activation, Coagulation System and IL-10 Signaling. The gene signatures in Proneural subtype are significantly enriched in pathways associated with cellular processes, such as Wnt/β-catenin Signaling and Cyclins and Cell Cycle Regulation. Signatures in the Neural subtype are significantly enriched in pathways associated with nervous system pathways and environmental information processing, such as nNOS Signaling in Skeletal Muscle Cells and cAMP-mediated signaling. Finally, the gene signatures in the Classical subtype are significantly enriched in pathways associated with the metabolism pathways, the nervous system and immune system, such as Fatty Acid Activation, CREB Signaling in Neurons, and PI3K Signaling in B Lymphocytes. They found the response to temozolomide in Classical and Mesenchymal subtypes was higher than that of neurotypes, and the Proneural subtype was lower than these three subtypes. They also developed a computational drug repurposing approach to predict GBM drugs based on the molecular subtypes. Protein kinase inhibitors, antipsychotics, and antidepressants have been identified as the most common drugs for all four subtypes. But in different subtypes, the ranking of drugs is different. In the Proneural subtype, antidepressants and antipsychotics were more effective. Anti-globulin inhibitors of the Mesenchymal subtype are involved in many immune system pathways and phenotypes. These results indicate that different molecular subtypes respond differently to drugs, and GBM subtype-specific therapies should be used.

Further evidence of molecularly guided treatment comes from Sandmann et al. that showed a 4.3 month increase in median survival with the addition of bevacizumab for IDH1 wild-type GBM in the proneural subgroup (Sandmann et al., 2015). The IDH1 R132H vaccine has been developed and shown promising results in animal models of IDH mutant glioblastomas (Schumacher et al., 2014; Dimitrov et al., 2015). These results demonstrate the necessity of diagnosing and developing personalized treatment plans according to IDH status.

Temozolomide is an oral alkylation agent. The main mechanism of temozolomide arrests the cell cycle at G2/M checkpoint, which leads to apoptosis of cancer cells (Alonso et al., 2007). The study showed the median survival was 12 months for patients receiving both temozolomide and radiation therapy and only 8 months for patients receiving radiation therapy alone (Alonso et al., 2007). However, in GBM, due to individual differences, the lack of MGMT methylation in some patients leads to the formation of temozolomide resistance. Herrlinger et al. found that for patients newly diagnosed, without MGMT methylation and with irinotecan/bevacizumab/radiation combination therapy had significantly prolonged mPFS (median progression – free survival) of 9.7 months. Temozolomide/radiation had significant mPFS of 5.9 months, an encouraging result that supports further investigation with this combination (Herrlinger et al., 2014). Therefore, before temozolomide treatment, it is advised to determine the methylation status of MGMT for most effective strategy.

Due to the heterogeneity of GBM, individualized treatment based on specific tumor subtype is clearly a more effective clinical strategy. Gene mutations in TP53, IDH-1 and PDGFR-A in Proneural subtype; mutations or amplification of EGFR gene in Classical subtype; NF-1 gene mutations in Mesenchymal subtype; and the expression of neural markers in Neural subtype are promising therapeutic targets. Several studies have shown that targeting these molecules improves treatment. For example, Sang et al. found the efficacy of SHP099, a potent, selective, and oral SHP-2 inhibitor for treating GBM with activated PDGFR-A signaling; and Liu et al. proved that the third-generation EGFR inhibitor osimertinib overcomes primary resistance by continuously blocking ERK signaling in GBM (Liu et al., 2019; Sang et al., 2019).

Due to subtype migration and molecular changes after recurrence, molecular evaluation of patients must be performed prior to chemotherapy. Patients undergoing surgical resection must undergo immunohistochemical studies to determine various predictors, such as MGMT methylation, to assist in treatment planning.

### Individualized Treatment

#### Gene Therapy

Gene therapy aims to introduce genetic material into cells to compensate for abnormal genes or to make beneficial proteins. If a mutated gene causes a necessary protein loss, gene therapy can introduce a normal gene to supplement the protein’s function. Gene therapy is the delivery of a gene through a vector to a cell. Viruses are often used as vectors because they can deliver new genes by infecting cells. These viruses are modified so that when they are used in humans, they do not cause disease. Adenovirus (AAV) vectors have been used to inject directly into GBM cells in the brain to express tumor-killing genes. Crommentujin et al. demonstrated the AAV9 vector, which produces the anticancer agent sTRAIL, killed up to 60% of GBM cells in mouse models and transfected cell lines (Gray et al., 2011; Crommentuijn et al., 2016). AAV9 virus vector is an excellent choice because its serotype can cross the blood-brain barrier during intravenous administration (Gray et al., 2011). CRISPR gene editing also belongs to gene therapy. By combining Cas9 nuclease with synthetic guide RNA and introducing it into the cell, the cell genome can be accurately trimmed, allowing existing genes to be removed or new ones added (Hendel et al., 2015). Using gene therapy technology to repair and compensate the tumor suppressor gene mutation in each subtype of GBM patients, such as PTEN mutation in the Classical subtype, may improve the survival time of patients.

#### Immunotherapy

Immunotherapy offers the promise of a sustained antitumor immunity that is pathway independent and has the potential to amplify antigens to boost immune responses. Peptide vaccines, such as EGFRvIII found in Classical GBM subtypes, can trigger immunity to GBM tumor cells expressing EGFRvIII. In a phase II trial involving 18 patients, EGFRvIII patients showed an overall survival of 26 months, compared with only 15 months for the control group (Heimberger, 2005). The vaccine has a promising future in immunotherapy for GBM. Tumor-specific antigen vaccines require confirmation that the tumor expresses the targeted antigen. Thus, immunotherapy limits the scope of these vaccines and the population in which they can be used, so specific vaccines can be designed according to the expression of molecules in different subtypes.

#### Organoid Model

Glioblastoma organs can be an effective model for rapid testing of personalized treatment strategies. The models allow researchers to reconstruct key features of a patient’s diseased brain to help paint a clearer picture of the cancer and then allow researchers to explore the best ways to treat it. Researchers have successfully transplanted eight glioblastoma organoids (GMOs) samples into brains of adult mice, administering standard care and targeted therapy to GBOs, including clinical trial drugs and chimeric antigen receptor T (CAR-T) cell immunotherapy (Jacob et al., 2020). For each treatment, the researchers showed that organ-like responses were different, and the effect was linked to genetic mutations in the patient’s tumor. The model opens the possibility of future clinical trials that can personalize treatment based on how a patient’s tumor responds to different drugs. Notably, the researchers have observed the benefits of treating organ-like organs with CAR-T therapy in clinical trials for EGFRvIII mutations, a driver of the disease. In 6 cases of GBOs, the EGFRvIII mutation was shown to have a specific effect on patient GBOs, with increased CART cells and decreased EGFRvIII expression cells (Jacob et al., 2020). These results highlight the potential of using personalized approaches to detect and treat glioblastoma.

## Conclusion

The unique and highly reproducible molecular changes discovered in recent years have begun to elucidate the diversity of GBM and contribute to the more effective classification of tumors. These studies provide insights into how to improve current treatment strategies. GBM genomics, transcription, and epigenetic features reveal critical molecular changes that may lead to pathologic disease progression. Large-scale analysis, like the TCGA project, confirm that GBM is a heterogenetic tumor at the molecular level that can be subdivided into different subtypes according to the molecular pathogenesis and biological entities of “driving factor” lesions. Although these comprehensive studies provide useful insights into the characteristics and classification of tumors, their limitations need to be considered when drawing conclusions. Some prognostic markers have appeared in these studies, and there is still a great need to identify true predictive markers to improve the treatment process of personalized care. In addition, the intra-tumoral heterogeneity of GBM needs to be further classified by single-cell sequencing technology to obtain a more complete and more precise inter-tumoral and intra-tumoral classification. We still need large-scale animal experiments and human clinical verification to improve treatment response and survival time among the different subtypes.

Through these molecular-level studies, we can further improve the molecular detection methods, guide the targeted therapy based on molecular classification, and form a set of accurate GBM molecular therapy manuals that can improve patient outcome.

## Author Contributions

QX and PZ contributed to the conception and design of manuscript. PZ drafted the manuscript. QX provided useful comments and suggestions. LD and QX revised the manuscript. All authors reviewed and approved the final manuscript.

## Conflict of Interest

The authors declare that the research was conducted in the absence of any commercial or financial relationships that could be construed as a potential conflict of interest.
